# The Involvement of the Mid1/Cch1/Yvc1 Calcium Channels in *Aspergillus fumigatus* Virulence

**DOI:** 10.1371/journal.pone.0103957

**Published:** 2014-08-01

**Authors:** Patrícia Alves de Castro, Jéssica Chiaratto, Lizziane K. Winkelströter, Vinícius Leite Pedro Bom, Leandra Naira Zambelli Ramalho, Maria Helena S. Goldman, Neil Andrew Brown, Gustavo H. Goldman

**Affiliations:** 1 Faculdade de Ciências Farmacêuticas de Ribeirão Preto, Universidade de São Paulo, Ribeirão Preto, Brazil; 2 Faculdade de Medicina de Ribeirão Preto, Universidade de São Paulo, Ribeirão Preto, Brazil; 3 Faculdade de Filosofia, Ciências e Letras de Ribeirão Preto, Universidade de São Paulo, Ribeirão Preto, Brazil; 4 National Laboratory of Science and Technology of Bioethanol (CTBE), Campinas, Brazil; University of Wisconsin - Madison, United States of America

## Abstract

*Aspergillus fumigatus* is a major opportunistic pathogen and allergen of mammals. Calcium homeostasis and signaling is essential for numerous biological processes and also influences *A. fumigatus* pathogenicity. The presented study characterized the function of the *A. fumigatus* homologues of three *Saccharomyces cerevisiae* calcium channels, voltage-gated Cch1, stretch-activated Mid1 and vacuolar Yvc1. The *A. fumigatus* calcium channels *cchA*, *midA* and *yvcA* were regulated at transcriptional level by increased calcium levels. The YvcA::GFP fusion protein localized to the vacuoles. Both *ΔcchA* and *ΔmidA* mutant strains showed reduced radial growth rate in nutrient-poor minimal media. Interestingly, this growth defect in the *ΔcchA* strain was rescued by the exogenous addition of CaCl_2_. The *ΔcchA*, *ΔmidA*, and *ΔcchA ΔmidA* strains were also sensitive to the oxidative stress inducer, paraquat. Restriction of external Ca^2+^ through the addition of the Ca^2+^-chelator EGTA impacted upon the growth of the *ΔcchA* and *ΔmidA* strains. All the *A. fumigatus ΔcchA*, *ΔmidA*, and *ΔyvcA* strains demonstrated attenuated virulence in a neutropenic murine model of invasive pulmonary aspergillosis. Infection with the parental strain resulted in a 100% mortality rate at 15 days post-infection, while the mortality rate of the *ΔcchA*, *ΔmidA*, and *ΔyvcA* strains after 15 days post-infection was only 25%. Collectively, this investigation strongly indicates that CchA, MidA, and YvcA play a role in *A. fumigatus* calcium homeostasis and virulence.

## Introduction

Calcium ions are universally important secondary messengers involved in numerous signaling pathways in all eukaryotic cells. Calcium-mediated signaling regulates a diverse array of biological processes including gene transcription, protein conformation, energy metabolism, endo- and exo-cytosis, cytoskeletal arrangement, and cell physiology [1], [2]. Additionally, in yeasts and filamentous fungi, Ca^2+^ is important for the regulation of cell cycle, sporulation, hyphal morphogenesis, hyphal orientation and pathogenesis [3]–[8]. Calcium homeostasis and signaling are based on the equilibrium between the accumulation and release of calcium from intracellular stores, such as the vacuole, endoplasmic reticulum (ER), mitochondria and golgi apparatus. In *Saccharomyces cerevisiae* free cytosolic Ca^2+^ concentrations are very low, but in response to external stimuli or stress, Ca^2+^ channels are opened and Ca^2+^ is rapidly mobilized from intracellular stores. Cytosolic Ca^2+^ binds to calmodulin that activates the phosphatase calcineurin, which in turn dephosphorylates the Crz1 transcription factor, resulting in its nuclear translocation, the modulation of gene transcription including calcium transporters, and the restoration of calcium homeostasis [9]–[12].

**Figure 1:**
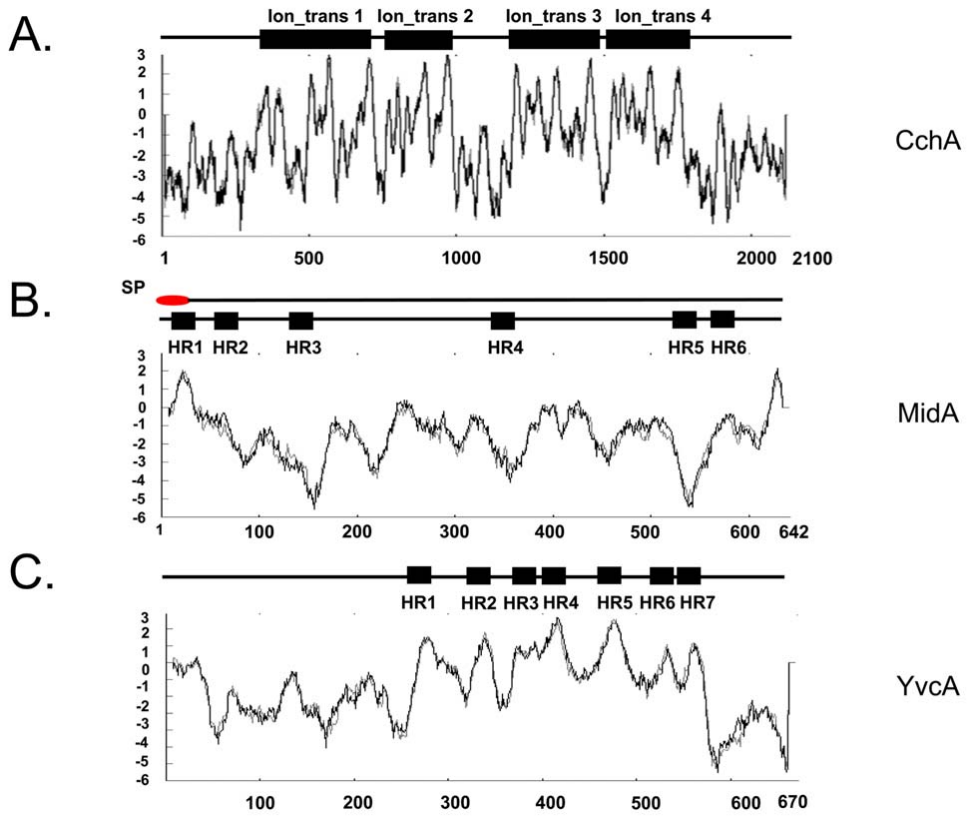
The identification of CchA, MidA, and YvcA orthologues in *A. fumigatus*. (A) CchA, (B) MidA, and (C) YvcA. Ion_trans 1 to 4 indicate four domains PF00520 (http://pfam.sanger.ac.uk/family/Ion_trans), each with six transmembrane-spanning regions. HR and SP indicate a hydrophobic regions and a signal peptide, respectively. The topologies were predicted using SMART database (http://smart.embl-heidelberg.de) and TMpred program (http://ch.embnet.org/software/TMPRED_form.html). The signal peptide was predicted by using SignalP 4.1 program (http://www.cbs.dtu.dk/services/SignalP/).

Calcium homeostasis is maintained by calcium channels, pumps and transporters. Calcium channels allow the passive flow of Ca^2+^ across cell membranes into the cytosol. Two major calcium uptake pathways including the high-affinity (HACS) and low-affinity (LACS) calcium uptake system have been identified in fungi [7], [8], [13]–[16]. HACS consists of the voltage-gated Ca^2+^ channel Cch1 [17], [18], the stretch-activated calcium channel/regulatory protein Mid1 [19] and the PMP22_Claudin superfamily member Ecm7 [14], [20]. Voltage-gated calcium channels sense the membrane potential and upon its depolarization open a gate allowing Ca^2+^ influx [1]. Stretch-activated channels are activated by cell deformations associated with contact sensing and thigmotropism [1]. HACS is the major route of calcium entry into the cell when availability is low [7], [8], [14]. Alternatively, LACS is active when calcium availability is high and consists of the Fig. 1, a pheromone-inducible plasma membrane protein [7], [21].

In *S. cerevisiae*, endoplasmic reticulum and environmental stresses as well as exposure to mating pheromone trigger a response of the HACS [7], [19], [22]–[24]. In filamentous fungi, HACS mutants demonstrate impaired calcium metabolism and reduced vegetative growth. In *A. nidulans*, CchA and MidA are important for the regulation of conidiation, hyphal polarity and cell wall components in low-calcium environments [8]. In phytopathogenic fungi, such as *Magnaporthe oryzae* and *Gibberella zeae*, Mid1 and Cch1 mutants have minor defects in growth and development, but they are still virulent [25]–[27]. However, the deletion of *mid1* in the *Claviceps purpurea* resulted in a complete loss of plant virulence [28]. Recent studies have indicated that in the human fungal pathogens *Candida albicans* and *Cryptococcus neoformans*, HACS was essential for sensing, and adapting to, the human host [8], [29]–[31]. These studies showed that HACS was required for the response to various stresses and also the tolerance of antifungal compounds, suggesting that Cch1 and Mid1 calcium channels are important in virulence and could represent targets for novel antifungal compounds.

The fungal vacuole is a major store for calcium. Fungal vacuolar calcium transporters, such as Ca^2+^ ATPases and Ca^2+^/H^+^ exchangers, are involved in removing Ca^2+^ ions from the cytosol and loading them into internal stores to avoid calcium toxicity [32]. In *S. cerevisiae*, *PMC1* is responsible for this process preventing growth inhibition by the activation of calcineurin in the presence of elevated calcium concentrations [33]. Conversely, a vacuolar membrane localized calcium channel, *YVC1*, which is a member of the transient receptor potential (TRP) family [34], [35] mediates calcium release from the vacuole in response to hypertonic shock [36], [37]. The null mutant of the *C. albicans YVC1* homologue showed a decreased stress response, altered morphogenesis and attenuated virulence [38], [39], suggesting that *ycv1* homologues may also play a role in pathogenicity. To date no functional analyses of *YVC1* homologues in filamentous fungi have been reported.

*Aspergillus fumigatus* is a major opportunistic pathogen and allergen of mammals [40], [41]. Multiple components of the calcium signaling pathway impact upon *A. fumigatus* virulence [6], [42]–[45]. In addition, three *PMC1* calcium transporter homologues in *A. fumigatus*, *pmc*A-C, were shown to be involved in calcium and manganese metabolism, but only *pmcA* influenced virulence [45]. Subsequently, the presented study characterized the *A. fumigatus* homologues of the *CCH1, MID1* and *YVC1* calcium channels. The phenotypes of the constructed *cchA, midA* and *yvcA* null mutants demonstrated their involvement in calcium metabolism and homeostasis. The *A. fumigatus cchA, midA*, and *yvcA* null mutants were avirulent in a murine model of invasive pulmonary aspergillosis. Therefore, HACS and the release of vacuolar calcium stores are essential for full virulence.

## Results

### Identification of *A. fumigatus* Mid1, Cch1 and Yvc1 homologues

In the *A. fumigatus* genome a single gene showed significant identity to each of the *S. cerevisiae* calcium channel encoding genes, *CCH1*, *MID1* and *YVC1*. These *A. fumigatus* gene were subsequently named *cchA* (Afu1g11110), *midA* (Afu5g05840) and *yvcA* (Afu3g13490). The predicted 2,125 amino acid sequence of *cchA* encoded four hydrophobic transmembrane domains (Figure 1A). The 642 amino acid protein of *midA* contained an N-terminal signal peptide and six putative transmembrane domains (Figure 1B). The 670 amino acid protein encoded by *yvcA* had seven putative transmembrane domains (Figure 1C). The overall topology of the three calcium channels in *A. fumigatus* was similar to that of Ca^2+^ voltage-gated channels in higher eukaryotes. The *A. fumigatus midA*, *cchA*, and *yvcA* are single copy genes and do not have paralogues (Figure S1 and Table S1). Moreover, they have very close homologues in other ascomycetes but more distantly related ones in *S. pombe*, *C. albicans*, and basidiomycetes (Figure S1).

**Figure 2:**
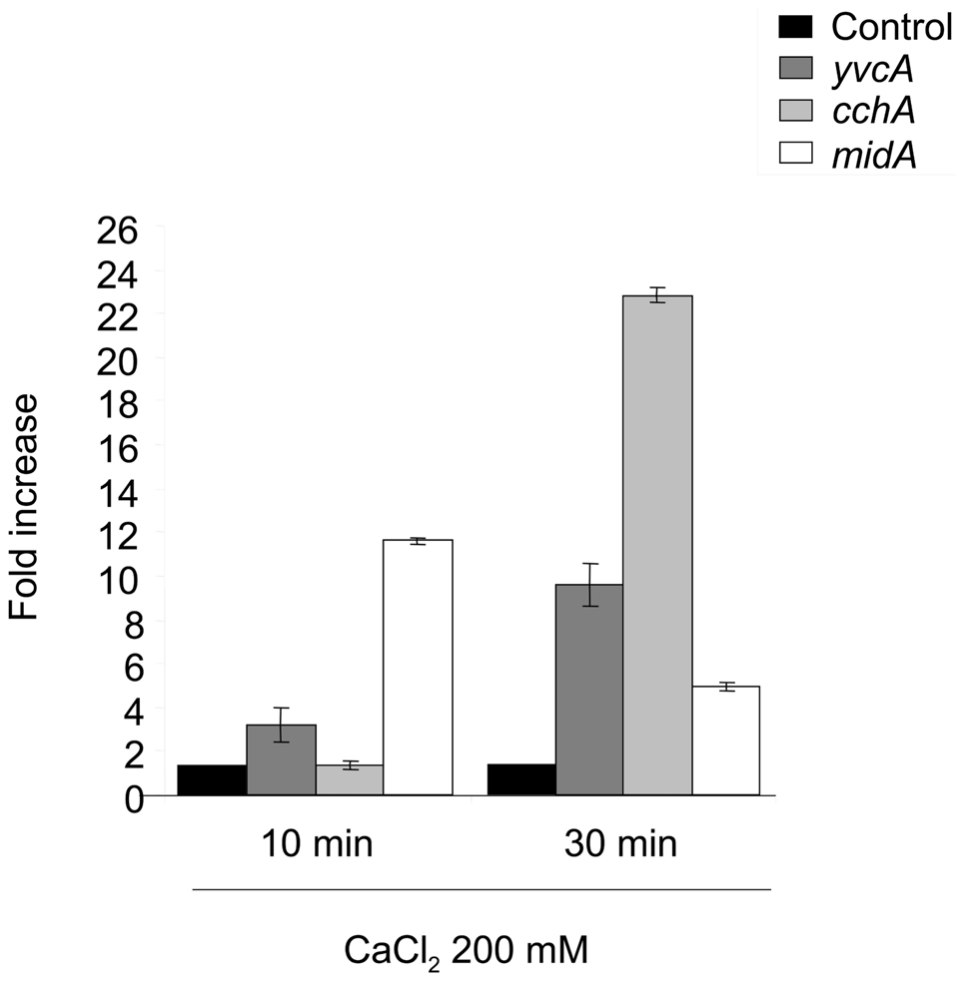
mRNA accumulation of the *A. fumigatus cchA*, *midA*, and *yvcA* genes. The wild-type and mutant strains were grown for 16 hours in YG medium at 37°C and the mycelia was either transferred to YG without any calcium for 30 minutes (Control:C) or for YG supplemented with 200 mM of calcium. RT-qPCR was used to quantify calcium channel mRNA abundance, which was normalized using β-tubulin (Afu1g10910). The relative quantitation of *cchA*, *midA*, *yvcA*, and β-tubulin was determined by comparison with a standard curve (C_T_ –values plotted against logarithm of the DNA copy number). The results presented are the means (± standard deviation) of four sets of experiments.

**Figure 3:**
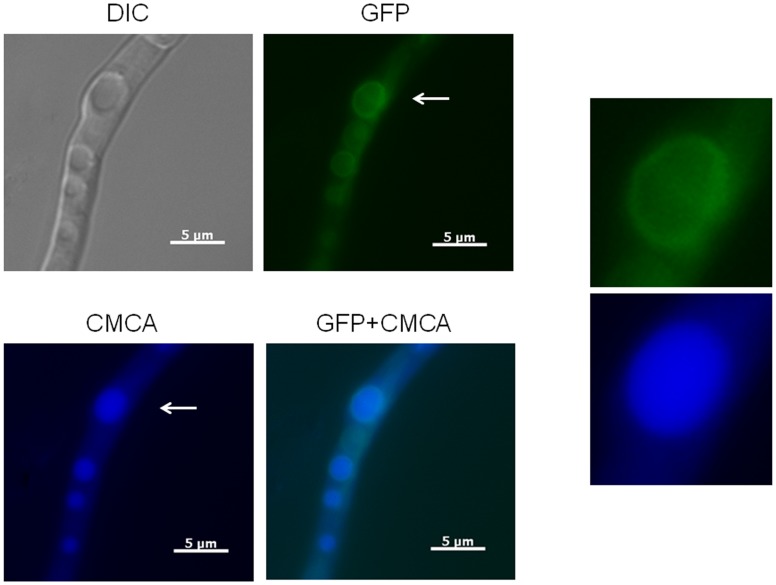
YvcA::GFP localizes to the vacuoles. Arrows indicate the vacuoles (insets). (DIC: Differential Interference Contrast; CMCA: CellTracker Blue). Bars, 5 µm.

To determine if the predicted calcium channels in *A. fumigatus* were influenced at the transcriptional level by calcium, *cchA*, *midA* and *yvcA* mRNA accumulation was assessed in the parental strain when exposed to 200 mM CaCl_2_. Calcium exposure for 10 and 30 minutes increased *yvcA*, *cchA*, and *midA* mRNA abundance by 3- and 9-fold, none and 22-fold, and 12- and 5-fold, respectively (Figure 2). These results suggest that *cchA*, *midA* and *yvcA* are regulated at transcriptional level by calcium. YvcA localization was assessed using a C-terminal GFP fusion protein. Growth of the YvcA::GFP strain resembled the parental strain under nutrient-rich and nutrient-poor conditions (data not shown), suggesting that the replacement of the wild-type gene with the GFP fusion construct in the transformed strain did not impair YvcA function. Within the hyphae the YvcA fusion protein localised to structures that resemble vacuoles during growth on minimal or complete media (Figure 3). The vacuolar identity of these structures was confirmed by staining the germlings with the vacuolar specific fluorophore CMCA (Figure 3, see insets). We were not able to see any increase or relocalization of the YvcA::GFP upon exposure to 200 mM of CaCl_2_ for 10 to 60 minutes (data not shown). These results strongly indicate YvcA is located on the vacuoles.

### Construction of the *A. fumigatus cchA, midA* and *yvcA* null mutants

**Figure 4:**
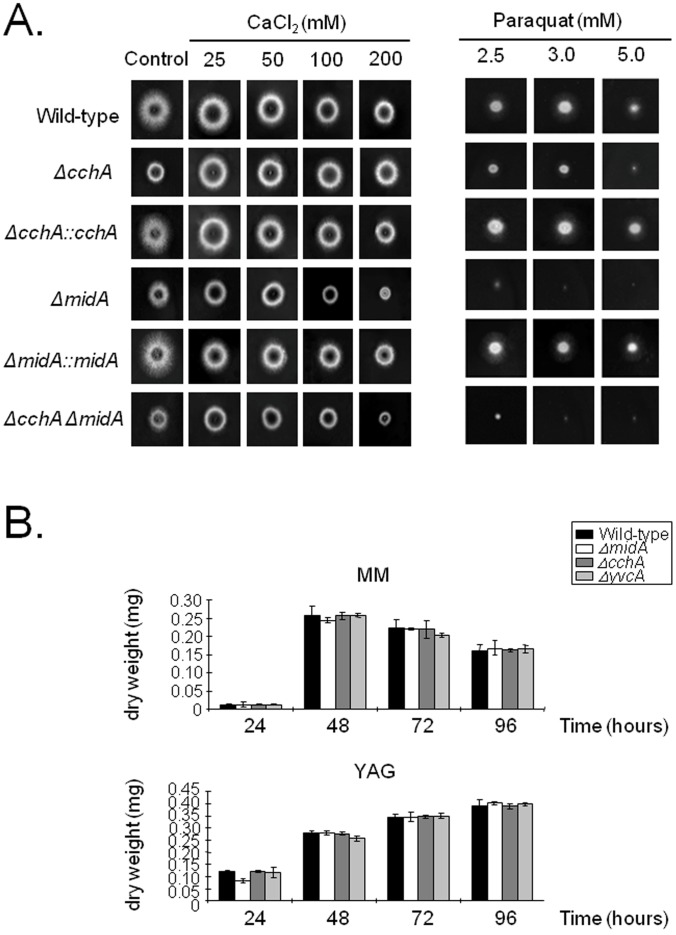
Growth of *A. fumigatus* parental and calcium channel mutant strains in different media. (A) The parental, *ΔcchA*, *ΔmidA*, *ΔcchA ΔmidA*, *ΔyvcA* and complementing strains in the presence or absence of calcium chloride or paraquat. (B) Dry weight (mg) of the parental, *ΔcchA*, *ΔmidA*, and *ΔyvcA* grown in liquid MM or YG cultures from 24 to 96 hours. The results are the means (± standard deviation) of three sets of experiments.

The function of *cchA*, *midA* and *yvcA* was investigated by generating the *ΔcchA*, *ΔmidA*, and *ΔyvcA* strains using a gene replacement strategy (Figure S2). In addition, a *ΔcchA ΔmidA* double mutant was created by inserting a deletion *midA::pyrG^+^* cassette into *ΔcchA pyrG^−^* mutant strain aiming to assess possible unique functions of each gene. The individual gene deletions were also complemented with the corresponding wild-type genes aiming to confirm the occurrence of possible secondary mutations during the construction of the deletion strains. The *ΔcchA*, *ΔmidA* and *ΔcchA ΔmidA* strains demonstrated reduced radial growth on solid MM, when compared to the parental strain (Figure 4A). Interestingly, the addition of different concentrations of CaCl_2_ rescued the growth defect of the *ΔcchA* (25 to 200 mM), *ΔmidA* (25 and 50 mM), and *ΔcchA ΔmidA* (25 and 50 mM) (Figure 4A). This positive CaCl_2_ effect reflects a compensation of lacking the calcium channels for suitable levels of intracellular calcium. All single, or double, gene deletion mutants and the respective complemented strains displayed parental-like responses to temperature, cell wall (congo red and calcofluor white), membrane (SDS), oxidative (*t*-butyl) and osmotic stress (NaCl and Sorbitol). However, the *ΔcchA*, *ΔmidA* and *ΔcchA ΔmidA* strains demonstrated increased sensitivity to the oxidative stress inducer, paraquat (Figure 4A). The *ΔyvcA* mutant showed no differential sensitivity to any of agents tested (data not shown). Curiously, no growth reduction was observed in liquid MM and YG media (Figure 4B). None of the three single gene deletions had an impact upon conidiation or germination rate (data not shown). The complemented *ΔchhA::cchA* ^+^, *ΔmidA::midA* ^+^ and *ΔyvcA::yvcA* ^+^ showed parental phenotypes, indicating that the observed phenotypes were due to the gene deletion(s) in the respective strains (Figure 4A).

**Figure 5:**
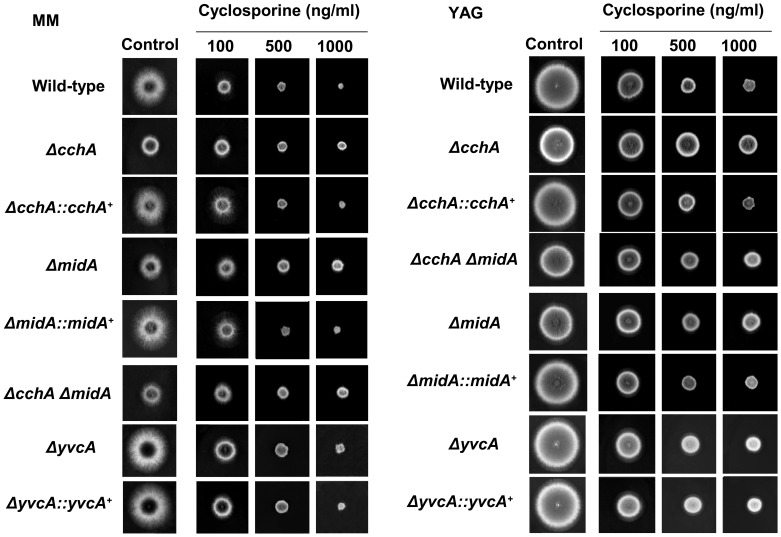
Growth of *A. fumigatus* parental, *ΔcchA*, *ΔmidA*, *ΔcchA ΔmidA*, *ΔyvcA* and complementing strains strains in MM and YAG supplemented with different cyclosporine concentrations.

**Figure 6:**
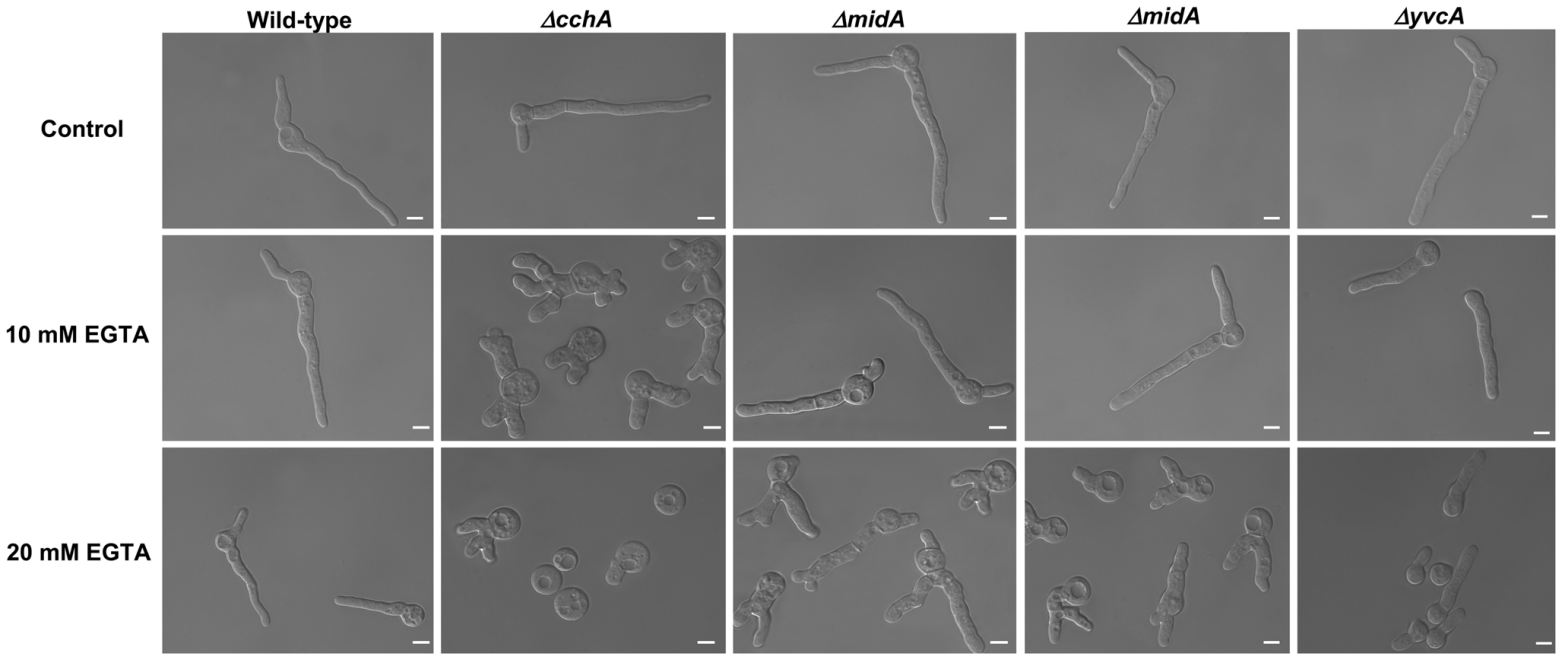
Restriction of external Ca^2+^ supply by the addition of Ca^2+^-chelating agent EGTA affects the growth of the *ΔcchA* and *ΔmidA* mutant strains. The parental, *ΔcchA*, *ΔmidA*, and *ΔcchAΔmidA* strains were grown in liquid MM supplemented with 0, 10 or 20 mM for 16 hours at 30°C. Bars, 5 µm.

Cyclosporine A (CsA) is an immunosuppressive drug that inhibits calcineurin signaling by forming a complex with the immunophilin, cyclophilin, which then inhibits calcineurin. The *ΔcchA*, *ΔmidA* and *ΔcchA ΔmidA* strains showed an increased resistance to cyclosporine in both MM and YAG; the *ΔyvcA* mutant was as sensitive as the wild-type strain (Figure 5). Growth in the presence of the Ca^2+^-chelating agent EGTA was subsequently utilized to assess the impact of the gene deletions during calcium limitation. Growth of the parental strain was not dramatically affected by the presence of 10 or 20 mM EGTA, while *ΔcchA* growth was restricted at 10 or 20 mM and *ΔmidA* growth was restricted at only 20 mM (Figure 6). As previous, the *ΔcchA ΔmidA* demonstrated a phenotype reminiscent of the *ΔmidA*. Interestingly, the presence of EGTA resulted in the *ΔcchA*, *ΔmidA*, and *ΔcchA ΔmidA* strains resembling the *A. fumigatus* calcineurin catalytic subunit deletion [6], *i.e.*, thicker swollen hyphae with dichotomous hyphal tips (Figure 6). Similar to the parental strain, EGTA had limited influence on the growth of the *ΔyvcA* mutant (Figure 6).

**Figure 7:**
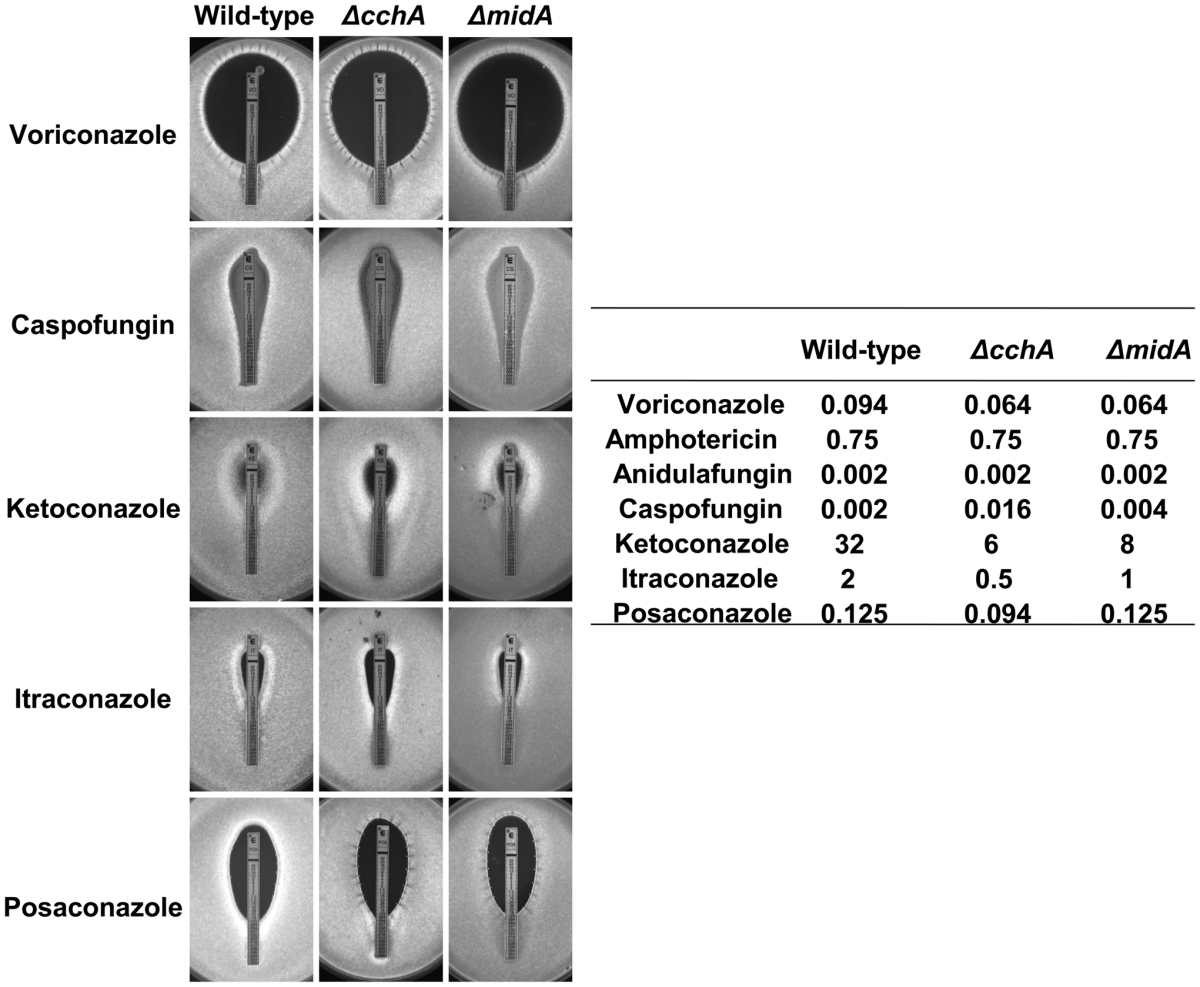
Susceptibility of *A. fumigatus* parental and calcium channel mutant strains to antifungal agents. YG agar plates were inoculated with 2×10^7^ conidia. Etest strips for voriconazole, amphotericin, Anidulafungin, ketoconazole, itraconazole, and posaconazole were overlaid on the media, and the plates were incubated at 37°C for 16–20 hours. Representative pictures showing the inhibition ellipses and the endpoint values obtained with the Etest strips are shown. Experiments with all antifungal drugs were performed in triplicate.

Calcium is an important cellular signal for fungal drug resistance [46]. The *ΔcchA* and *ΔmidA* strains were more susceptible to various fungicides including voriconazole, ketoconazole, and itraconazole, while *ΔcchA* was also more susceptible to posaconazole (Figure 7). In contrast, both the *ΔcchA* and *ΔmidA* strains were more resistant to caspofungin (Figure 7). The *ΔyvcA* mutant showed no difference in susceptibility, from the parental strain, to any of the antifungal agents tested (data not shown).

**Figure 8:**
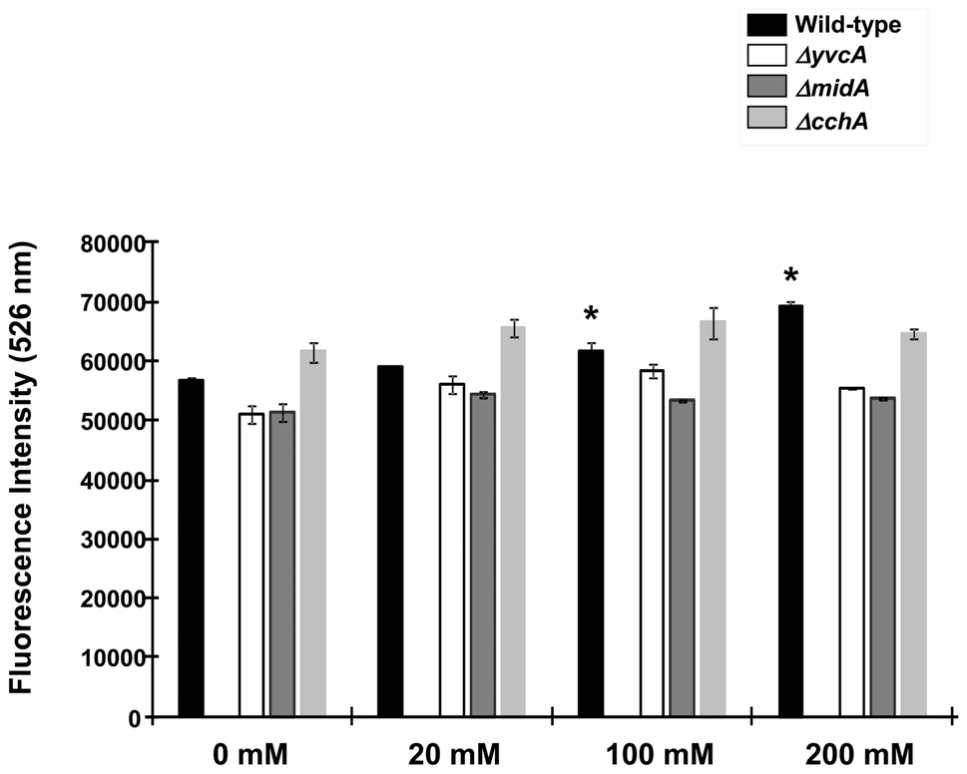
The *ΔcchA*, *ΔmidA*, and *ΔyvcA* strains have decreased accumulation of calcium in the cytoplasm. The relative levels of intracellular calcium in the parental, *ΔcchA*, *ΔmidA*, and *ΔyvcA* mutant strains were determined using the calcium-sensitive dye Fluo-3. The relative Ca^2+^ concentration was determined based on the fluorescence ratio after dual-wavelength excitation (fluorescent intensity at 526 nm [FI506 nm]/[FI526 nm]. Data shown are means of three repetitions (± standard deviations). Statistical analysis was performed by using *t*-test (*, *p*<0.01) comparing the treatments at 20, 100, and 200 mM to the 0 mM CaCl_2_.

The concentration of free calcium within the cell was evaluated in the *A. fumigatus* parental, *ΔcchA*, *ΔmidA*, and *ΔyvcA* mutant strains using Fluo-3 (Invitrogen), which is a highly sensitive fluorescent dye for the rapid measurement of calcium. Fluo-3 passively diffuses across cell membranes. Within the cell, the esters are cleaved by intracellular esterases yielding a fluorescent indicator that can no longer diffuse across membrane. Upon Ca^2+^-binding, Fluo-3 exhibits an absorption shift from 506 to 526 nm. Thus, the relative intracellular Ca^2+^ concentration was evaluated based upon the fluorescence ratio after dual-wavelength excitation. During the exposure to extracellular calcium concentrations from 0, 100, and 200 mM, in the parental strain there was an increase in intracellular calcium (Figure 8). The *ΔyvcA*, *ΔcchA* and *ΔmidA* strains showed a constant low level of intracellular calcium. Alternatively, the *ΔcchA* strain demonstrated higher intracellular calcium than the parental strain when in the presence of 0, 20, and 100 mM extracellular calcium (Figure 8). Collectively, these results suggest that CchA and MidA play an important role in the calcium uptake while YvcA is important for removing high calcium concentrations from the cytoplasm.

Recently, *C. albicans* ability to adhere to a polystyrene surface and buccal epithelial cells was inhibited by exposure to verapamil, a calcium channel blocker [47]. Biofilm formation on polystyrene was significantly inhibited about 25 to 30% in the *ΔmidA* and *ΔyvcA* mutant strains when measured by crystal violet staining (Figure S3). These results were further confirmed by a reduction of about 20% biomass in both strains when grown in static growth on polystyrene Petri dishes (Figure S3). As previously shown, all the mutant strains have growth comparable to the wild-type strain (Figures 4B and S3).

### The *A. fumigatus ΔcchA, ΔmidA* and *ΔyvcA* mutant strains are avirulent in a low dose murine infection model

**Figure 9:**
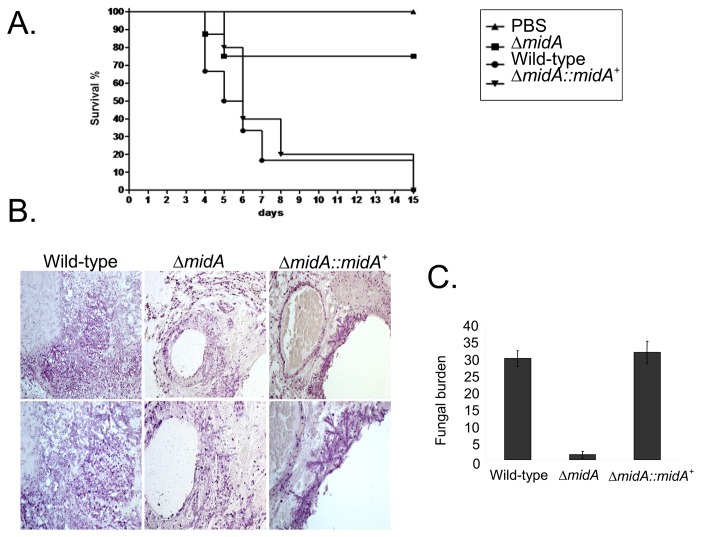
*A. fumigatus cchA* contributes to virulence in neutropenic mice. (A) Comparative analysis of parental, *ΔcchA*, and *ΔcchA::cchA* ^+^ strains in a neutropenic murine model of pulmonary aspergillosis. A group of 10 mice per strain was intranasally infected with a 20 µl suspension of conidiospores at a dose of 5.0×10^4^. (B) Histological analysis of infected murine lung was performed 72 hours after infection with the wild-type strain and it reveals invasion of the murine lung epithelium. (C) Fungal burden was determined 48 hours post-infection by real-time qPCR based on 18S rRNA gene of *A. fumigatus* and an intronic region of the mouse GAPDH gene. Fungal and mouse DNA quantities were obtained from the Ct values from an appropriate standard curve. Fungal burden was determined through the ratio between ng of fungal DNA and mg of mouse DNA. The results are the means (± standard deviation) of five lungs for each treatment. Statistical analysis was performed by using *t*-test (*, *p*<0.01).

**Figure 10:**
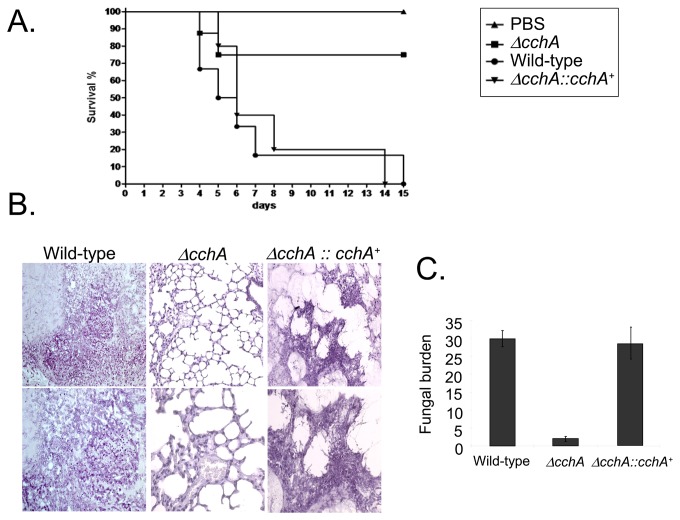
*A. fumigatus midA* contributes to virulence in neutropenic mice. (A) Comparative analysis of parental, *ΔmidA*, and *ΔmidA::midA* ^+^ strains in a neutropenic murine model of pulmonary aspergillosis. Experimental details are described in Figure 8A. (B) Histological analysis of infected murine lung was performed 72 hours after infection with the wild-type strain and it reveals invasion of the murine lung epithelium. (C) Fungal burden was determined 48 hours post-infection by real-time qPCR based on 18S rRNA gene of *A. fumigatus* and an intronic region of the mouse GAPDH gene. Experimental details are described in Figure 8C.

**Figure 11:**
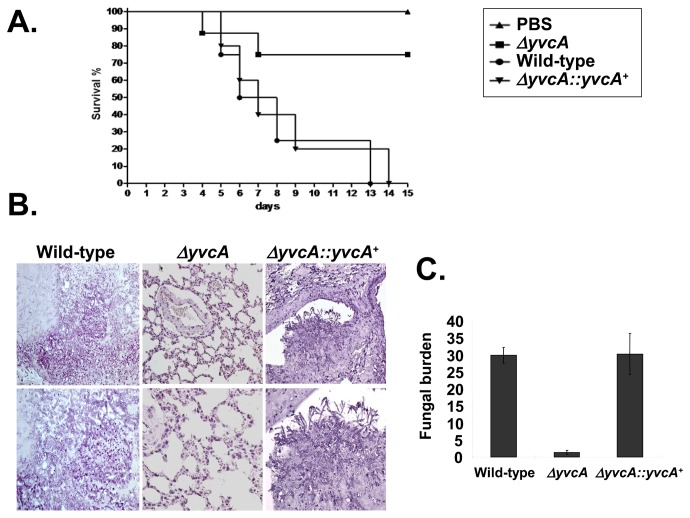
*A. fumigatus yvcA* contributes to virulence in neutropenic mice. (A) Comparative analysis of parental, *ΔyvcA*, and *ΔyvcA::yvcA* ^+^ strains in a neutropenic murine model of pulmonary aspergillosis. Experimental details are described in Figure 8A. (B) Histological analysis of infected murine lung was performed 72 hours after infection with the wild-type strain and it reveals invasion of the murine lung epithelium. (C) Fungal burden was determined 48 hours post-infection by real-time qPCR based on 18S rRNA gene of *A. fumigatus* and an intronic region of the mouse GAPDH gene. Experimental details are described in Figure 8C.

The involvement of CchA, MidA and YvcA in *A. fumigatus* pathogenicity was assessed in a neutropenic murine model of invasive pulmonary aspergillosis. Infection with the parental strain resulted in 100% mortality at 15 days post-infection, while *ΔcchA*, *ΔmidA*, and *ΔyvcA* infection resulted in a significant reduction (*p*<0.005) in mortality rate, with approximately 25% mortality after 15 days (Figures 9A, 10A and 11A). Full virulence was restored in independent strains resulting from the single ectopic reintegration of the parental *cchA*, *midA*, and *yvcA* gene (Figure S2) and the complemented strains (Figures 9A, 10A, and 11A), directly linking the attenuation of virulence to the disruption of three calcium channels.

Histopathological examinations of the parental, *ΔcchA*, *ΔmidA* and *ΔyvcA* infected tissues were performed to identify the differences in growth rate, tissue invasion and the inflammatory response. After 72 hours post-infection the lungs of mice infected with the parental strain contained multiple sites of invasive hyphal growth that penetrated the pulmonary epithelium in major airways (Figures 9B, 10B, and 11B) and formed pockets of branched invading hyphae originating from the alveoli (Figure 9B, 10B, and 11B). In contrast, *ΔcchA*, *ΔmidA*, and *ΔyvcA* infections typically contained inflammatory infiltrates in bronchioles, some of which contained poorly germinated or ungerminated conidia (Figures 9B, 10B, and 11B). Fungal burden measured by real-time PCR showed that the growth of the *ΔcchA*, *ΔmidA* and *ΔyvcA* strains within the lungs was less than the parental and complemented strains (Figures 9C, 10C, and 11C, *p*<0.0001). This data strongly indicates that CchA, MidA, and YvcA play a role in *A. fumigatus* virulence.

## Discussion

Calcium homeostasis and signaling is essential for numerous biological processes and importantly impacts upon the growth, stress tolerance and virulence of fungal pathogens [1], [48], [49]. Several elements of the calcium signal transduction machinery sense and transport Ca^2+^, which in turn translate the spatiotemporal fluctuation in Ca^2+^ levels into a cellular response [48]. The filamentous ascomycete *A. fumigatus* is an opportunistic pathogen and major allergen of mammals [40], [41]. Multiple components of the calcium signaling cascade have previously been shown to impact upon *A. fumigatus* calcium homeostasis and virulence [6], [33], [42], [49]–[53]. For example, the calcineurin catalytic subunit *calA* deficient strain had severe defects in hyphal extension, branching and conidial architecture [6], while the downstream calcineurin-activated transcription factor CrzA directly controlled the transcription of calcium transporters *pmcA-C* [45]. Significantly, all the *A. fumigatus ΔcalA*, *ΔcrzA*, and *ΔpmcA* mutants were avirulent in the murine model of invasive pulmonary aspergillosis [6], [42], [45]. Generally, cytosolic Ca^2+^ concentrations are very low (50 to 100 nM). Calcium channels, pumps, and transporters are essential to control intracellular calcium levels. However, how *A. fumigatus* uptakes and stores calcium is unclear. Ca^2+^ influx and efflux channels such as Mid1, Cch1, and Yvc1 from *S. cerevisiae* have been identified in several filamentous fungi and linked to pathogenesis [54], [55]. Therefore, the function of three calcium channels in maintaining Ca^2+^ homeostasis during *A. fumigatus* infection was investigated. Growth in the presence of calcium-chelator EGTA represents calcium limiting conditions that would require HACS. The *ΔcchA* and Δ*midA* strains demonstrated growth defects reminiscent of *ΔcalA* during growth under such conditions, strongly indicating that *A. fumigatus* MidA and CchA are important for calcium uptake in low-calcium environments. Moreover, the accumulation of intracellular calcium was lower in the Δ*midA* and Δ*cchA* strains, while the fact that Δ*cchA* and Δ*midA* were also more resistant to cyclosporine, suggests a low level of calcineurin activity and implies that intracellular calcium level were low. The exogenous supply of high extracellular calcium concentrations restored the growth defect of Δ*cchA* and Δ*midA* strain.

In *S. cerevisiae*, Mid1 is located in the plasma membrane as well as in the ER membrane [56], while in *Botrytis cinerea* the Mid1 homologue is located to the ER membrane and nuclear envelope [16]. The interaction between Mid1 and Cch1 has been demonstrated in *S. cerevisiae* by co-immunoprecipitation [57], [58] and in *A. nidulans* using the yeast two-hybrid system [8]. However, in *B. cinerea* a split ubiquitin-based yeast two-hybrid approach did not reveal any interaction between Mid1 and Cch1 [16]. Although Cch1 and Mid1 have been shown to physically interact in various fungal systems, it remains to be addressed if *A. fumigatus* MidA and CchA physically interact. The corresponding individual *A. fumigatus midA* and *cchA* null mutants showed complementing and contrasting phenotypes, resulting in the hypothesis of unique gene functions for either Cch1 or Mid1. This was observed in several species such as *S. cerevisiae* [17], [18], [59], *C. albicans* [60], *A. nidulans* [8], and *B. cinerea* [16].

Both the *A. fumigatus ΔcchA* and *ΔmidA* mutants were more sensitive to oxidative stress. Peiter *et al.* [23] demonstrated that the *S. cerevisiae Δcch1 Δmid1* double mutant was hypersensitive to iron, that iron stress led to increased oxidative stress and that Cch1p-Mid1p was essential to tolerate this condition. The *C. albicans* homologue Ecm7 is an integral protein membrane with a role in HACS, which is also involved in the oxidative stress response and hyphal development [20]. During the aging of mammalian cells, Ca^2+^ signaling is altered and excess ROS can be produced due to an ineffective mitochondrial respiration [61]. It could be speculated that the impairment of calcium homeostasis, caused by deficiencies in the calcium uptake system, affect mitochondrial function and in turn cause oxidative stress [62].

The *A. fumigatus* Δ*cchA* and Δ*midA* strains demonstrated attenuated virulence in a murine model of pulmonary aspergillosis. In other human pathogenic fungi, such as *C. albicans* and *C. neoformans*, the virulence of *CCH1* and *MID1* null mutants was attenuated in the respective mouse infection models [29], [60]. In phytopathogenic fungi, such as *C. purpurea* and *G. zeae*, the calcium channel mutants respectively were non-pathogenic or showed slower disease progression [26]–[28]. In contrast, in *B. cinerea* deletion of Cch1 and Mid1 did not impact on virulence [16]. The observed difference in the impact of Cch1 and Mid1 deletion in the aforementioned phytopathogens could reflect differences in infection strategy. As the biotroph *C. purpurea* and hemibiotroph *G. zeae* both colonize live host tissues to establish infection, possibly experiencing calcium limiting conditions, while the necrotroph *B. cinerea* rapidly induces host cell death ahead of infection and may not rely on the HACS. Recently, Jiang *et al.* [63] characterized the *A. fumigatus* Mid1 homologue. The phenotypes shown for the *afmid1* null mutant mostly agreed with what was observed in the presented study. However, Jiang and colleagues [63] showed that the deletion of *afmid1*, referred to as Δ*midA* in the current investigation, led to hypervirulence in the immunosuppressed mice model. The contrasting results observed by these authors concerning *afmid1* virulence could be due to the different genetic backgrounds of the strains used. However, the reason for this discrepancy in virulence remains to be investigated.

The *S. cerevisiae YVC1* vacuolar calcium channel mediates the release of Ca^2+^ from the vacuole in response to hyperosmotic stress [34], [36]. The investigation of the *A. fumigatus yvcA* represents the first *YVC1* homologue to be functionally characterized in filamentous fungi. Krishnan *et al.* [64] recently showed that a novel mRNA isoform of *yvcA* was induced in the *A. fumigatus* ER translatome during ER stress, in an unfolded protein response-dependent manner. As predicted due to its function in *S. cerevisiae*, the *A. fumigatus* YvcA located to the vacuolar membrane. Surprisingly, no phenotype except for the decrease in the adhesion/biofilm formation and an attenuated virulence, was observed for the *A. fumigatus yvcA* null mutant. It is possible we have not observed any phenotype in the *ΔyvcA* mutant because there is a functional redundancy between *A. fumigatus* YvcA and PmcA. Actually, we have observed PmcA is located at the cell and vacuolar membranes (de Castro, unpublished results).

In conclusion, the *A. fumigatus* calcium channels MidA, CchA, and YvcA are all required for full virulence in an animal infection model. This investigation implies that CchA and MidA participate in the *A. fumigatus* Ca^2+^-calcineurin signaling pathway, influencing intracellular calcium homeostasis. Further studies are necessary to address the role of YvcA and how calcium homeostasis and oxidative stress are regulated by CchA and MidA.

## Materials and Methods

### Ethics statement

The principles that guide our studies are based on the Declaration of Animal Rights ratified by the UNESCO in January 27, 1978 in its articles 8th and 14th. All protocols used in this study were approved by the local ethics committee for animal experiments from the Campus of Ribeirão Preto from Universidade de São Paulo (Permit Number: 08.1.1277.53.6; studies on the interaction of *Aspergillus fumigatus* with animals). All animals were housed in groups of five within individually ventilated cages and were cared for in strict accordance with the principles outlined by the Brazilian College of Animal Experimentation (Princípios Éticos na Experimentação Animal - Colégio Brasileiro de Experimentação Animal, COBEA) and Guiding Principles for Research Involving Animals and Human Beings, American Physiological Society. All efforts were made to minimize suffering. Animals were clinically monitored at least twice daily and humanely sacrificed if moribund (defined by lethargy, dyspnoea, hypothermia and weight loss). All stressed animals were sacrificed by cervical dislocation. File S1 reports all the ARRIVE checklist for reporting animal studies.

### Strains, media and culture methods

The *A. fumigatus* strains used in this study were CEA17 (*pyrG* ^−^) and CEA17- 80 (as the parental in all the experiments). The media used were: complete medium composed of 2% w/v glucose, 0.5% w/v yeast extract, trace elements (YAG) or YUU (YAG supplemented with 1.2 g of uracil and uridine) and minimal medium (MM) composed of 1% glucose or 2% glycerol, trace elements and nitrate salts [65], pH 6.5. Solid versions of the aforementioned media were prepared via the addition of 2% w/v agar. Strains were grown at 37°C.

### Construction of the *A. fumigatus* mutants

The cassettes for gene replacement were constructed by *in vivo* recombination in *S. cerevisiae* as previously described by Colot *et al.* [66]. Approximately 1.5 kb from the 5′-untranslated region (UTR) and 3′-UTR flanking region of the targeted genes were selected for primer design. The primers 5F and 3R contained a short sequence homologous to the multiple cloning site (MCS) of the pRS426 plasmid. Both the 5- and 3-UTR fragments were PCR-amplified from *A. fumigatus* genomic DNA (gDNA). The *pyrG* inserted into the gene replacement cassettes was amplified from pCDA21 plasmid and was used to generate a marker for prototrophy in the mutant strains. Each fragment along with the *Bam*HI/*Eco*RI cut pRS426 plasmid were transformed into the *S. cerevisiae* strain SC94721 using the lithium acetate method [67]. The transformant DNA was extracted according to Goldman, *et al.* [68]. The cassette was PCR-amplified from the plasmids utilizing TaKaRa Ex Taq DNA Polymerase (Clontech Takara Bio) and used for *A. fumigatus* transformation. Southern blot analyses were used to confirm a single homologous integration event at the targeted *A. fumigatus* locus. The deleted mutant strains were complemented by co-transforming the respective gene plus a 1 kb flanking regions together with the pHATα vector [69] and selecting for hygromycin resistance on MM containing 150 mg/ml of hygromycin B.

The C-terminal Yvc::GFP fusion protein was created by cloning the *yvc* ORF (minus the stop codon) in frame with the green fluorescent protein (GFP) gene. The *gfp* and *yvc* sequences were separated by four additional codons that after translation produce a four amino acid linker (glycine-threonine-arginine-glycine) [70]. First, the *yvc* ORF plus 500 bp of the 5′-UTR were amplified from gDNA of the wild-type strain using the primers yvc pRS426 5Fw and yvc SPACER GFP Rv. The GFP ORF was amplified from the pMCB17apx plasmid (provided by Vladimir P. Efimov) using primers Spacer GFP Fw and GFP VE3′ AF. The selective marker *pyrG* was PCR amplified from the pCDA21 plasmid using the primers GFP pyrG Fw and pyrG Rv. Approximately 600 bp of the 3′-UTR were amplified using the primers Afu yvc 3Fw and Afu yvc 3Rv primers. As describe previously, the *S. cerevisiae in vivo* recombination system was used for production of the transformation cassette. The PCR-amplified cassette was transformed into the *A. fumigatus* wild-type strain. The primers used above are described in Table S2.

### Phenotypic assays

The E-tests (Biomérieux) were performed according to the manufacturer's instructions as follows, 1×10^7^ conidia were inoculated into 20 ml YAG medium containing half the concentration of agar. E-test strips were placed on the surface. Plates were incubated at 37°C for 24 h. The minimum inhibitory concentration was determined by analyzing the growth inhibition formed around the strip of voriconazole, amphotericin B, caspofungin, ketoconazole, itraconazole, posaconazole, anidulafungin.

### YvcA::GFP microscopy

YVC::GFP conidia were grown on coverslips in 4 ml of MM for 16 h at 30°C. After incubation, the coverslips with adherent germlings were left untreated or treated with CaCl_2_ (200 mM) for 10, 30 and 60 min. CMAC (CellTracker Blue CMAC - Molecular Probes) staining of vacuoles was performed as described previously [71]. After growth, 10 µM CMAC dye was added to the cultures for 15 min, at 30°C. Subsequently, the coverslips were rinsed with PBS and mounted for examination. Slides were visualized on a Carl Zeiss Observer Z1 fluorescence microscope using the excitation wavelength of 450 to 490 nm, and emission wavelength of 500 to 550 nm. DIC (differential interference contrast) images and fluorescent images were captured with an AxioCam camera (Carl Zeiss) and processed using AxioVision software (version 4.8).

### Maintenance of intracellular calcium homeostasis investigation

A fluorescent calcium indicator (Fluo-3, AM; Molecular Probes) was used to investigate the maintenance of intracellular calcium homeostasis. For this 1×10^7^ conidia were added to 15 ml MM and incubated for 5 hours at 37°C. A minimum of 3 replicates was performed for each strain. After incubation, the medium was discarded by centrifugation and the samples were resuspended in 2 ml PBS. Then, 200 µl of this cell suspension were placed in a 96-well, black/clear, flat bottom microtitre plate (BD Falcon) and a solution of CaCl_2_ (20, 100, or 200 mM) containing 10 µM Fluo-3 was added. Plates were incubated for 10 min at 30°C. Control samples were incubated for 10 min with Fluo-3 without calcium treatment. The samples were washed again with PBS and fluorescence at 526 nm determined on a SpectraMax i3 spectrometer (Molecular Devices).

### Crystal violet assay and biofilm formation

Crystal violet assays were used to assess biofilm formation as described previously by Mowat *et al* [72]. *A. fumigatus* biofilms were formed in 96-well microtitre plates (Corning® Costar®). Biofilms were formed by inoculating 1×10^4^ conidia/ml in MM containing 0.1% glucose. The plates were incubated at 37°C for 16 hours. Subsequently, the media was discarded and the biofilms were carefully washed three times with sterile PBS remove non-adherent cells. The biofilms were then dried at 60°C for 10 min and 200 µl of 0.5% crystal violet (Sigma) solution added. The plate was then incubated at room temperature for 5 min and then extensively washed with distilled water until all unbound crystal violet was removed. The plate was dried again and then 200 µl ethanol was added to each well to elute the bound crystal violet. The ethanol was then transferred to a clean 96-well microtitre plate and the absorbance read at 570 nm using a SpectraMax i3 spectrometer (Molecular Devices). A minimum of 6 replicates was performed for each strain.

In addition, *A. fumigatus* biofilm biomass was determined as described by Shopova *et al.* [73]. Briefly, conidial suspensions of 1×10^3^ to 1×10^6^ conidia/per well were inoculated in HEPES-buffered RPMI 1640 with L-glutamine. This suspension was inoculated into pre-sterilized polystyrene petri dish (static growth) or glass flasks (planktonic growth) and grown for 96 hours at 37°C. Biomass was determined by dry weight.

### RNA extraction and real-time PCR reactions

Mycelia were harvested by filtration, washed twice with H_2_O and immediately frozen in liquid nitrogen. For total RNA extractions, the mycelia were ground in liquid nitrogen with pestle and mortar. Total RNA was extracted with Trizol (Invitrogen, USA). The integrity of the RNA from each treatment was assessed using an Agilent 2100 Bioanalyzer. RNase-free DNase I treatment was carried out as previously described by Semighini *et al.* [74]. Twenty micrograms of total RNA was treated with DNase I, purified using the RNAeasy kit (Qiagen) and cDNA synthesized using the SuperScript III First Strand Synthesis system (Invitrogen) with oligo(dT) primers, according to the manufacturer's instructions. All the qPCR reactions were performed using an ABI 7500 Fast Real-Time PCR System (Applied Biosystems, USA) and SYBR Green PCR Master Mix (Applied Biosystems, USA). The reactions and calculations were performed according to Semighini et al. [74]. The primers used are described in Table S2.

### Murine model of pulmonary aspergillosis, lung histopathology and fungal burden

The murine model of pulmonary aspergillosis was performed according to Dinamarco, *et al.* [45]. Outbreed female mice (BALB/c strain; body weight, 20 to 22 g) were housed in vented cages containing 5 animals. Mice were immunosuppressed with cyclophosphamide, which was administered intraperitoneally (150 mg/kg of body weight) on days -4, -1 and 2 prior to and post infection. Hydrocortisonacetate (200 mg/kg) was injected subcutaneously on day -3. Fresh *A. fumigatus* conidia were grown on YAG for 2 days prior to infection, then harvested in PBS and filtered through Miracloth (Calbiochem). Conidial suspensions were spun for 5 min at 3,000× *g*, washed three times with PBS, counted using a hemocytometer, and resuspended at a concentration of 2.5×10^6^ conidia/ml. The viability of the administered inocula were confirmed by incubating a serial dilution of the conidial suspension, on YAG media, at 37°C. Mice were anesthetized by halothane inhalation and infected by intranasal instillation of 5.0×10^4^ conidia in 20 µl of PBS. As a negative control, a group of 5 mice received PBS only. Mice were weighed every 24 h from the day of infection and visually inspected twice daily. In the majority of cases, the endpoint for survival experimentation was identified when a 20% reduction in body weight was recorded, at which time the mice were sacrificed. The statistical significance of comparative survival values was calculated using log rank analysis and the Prism statistical package. Additionally, at 3 days post-infection, 2 mice per strain were sacrificed and the lungs were removed, fixed, and processed for histological analysis.

### Lung histopathology and fungal burden

After sacrifice, the lungs were removed and fixed for 24 hours in 3.7% formaldehyde–PBS. Samples were washed several times in 70% alcohol before dehydration in a series of alcohol solutions of increasing concentrations. Finally, the samples were diafanized in xylol and embedded in paraffin. For each sample, sequential 5-µm-thick sections were collected on glass slides and stained with Gomori methenamine silver (GMS) or hematoxylin and eosin (HE) stain following standard protocols [40]. Briefly, sections were deparaffinized, oxidized with 4% chromic acid, stained with methenamine silver solution, and counterstained with picric acid. For HE staining, sections were deparaffinized and stained first with hematoxylin and then with eosin. All stained slides were immediately washed, preserved with mounting medium, and sealed with a coverslip. Microscopic analyses were done using an Axioplan 2 imaging microscope (Carl Zeiss) at the stated magnifications under bright-field conditions.

To investigate fungal burden in murine lungs, mice were infected as described previously, but with a higher inoculum of 1×10^6^ conidia/20 µl. The higher inoculum, in comparison to the survival experiments, was used to increase fungal DNA detection. Animals were sacrificed 72 h post-infection, and both lungs were harvested and immediately frozen in liquid nitrogen. Samples were homogenized by vortexing with glass beads for 10 min, and DNA was extracted and the subsequent use of the phenol-chloroform method. DNA quantity and quality were assessed using a NanoDrop 2000 spectrophotometer (Thermo Scientific). At least 500 µg of total DNA from each sample was used for quantitative real-time PCRs. A primer and a Lux probe (Invitrogen) were used to amplify the 18S rRNA region of *A. fumigatus* (primer, 5′-CTTAAATAGCCCGGTCCGCATT-3′; probe, 5′-CATCACAGACCTGTTATTGCCG-3′) and an intronic region of mouse GAPDH (glyceraldehyde-3-phosphate dehydrogenase) (primer, 5′-CGAGGGACTTGGAGGACACAG-3′; probe, 5′-GGGCAAGGCTAAAGGTCAGCG-3′). Six-point standard curves were calculated using serial dilutions of gDNA from all the *A. fumigatus* strains used and the uninfected mouse lung. Fungal and mouse DNA quantities were obtained from the threshold cycle (*CT*) values from an appropriate standard curve. Fungal burden was determined as the ratio between picograms of fungal and micrograms of mouse DNA.

## Supporting Information

Figure S1 **Phylogenetic reconstruction by using “One Click” (** http://www.phylogeny.fr **) for (A) MidA and (C) YvcA and Mega [Maximum Likelihood using Jones-Taylor-Thornton (JTT) model (Nearest-Neighbor-Interchange, NNI)] for (B) CchA.** The accession numbers for ll the proteins used for alignment are described in Table S1.
(PPTX)

Figure S2 (A) Schematic representation of the deletion cassette assembly. Southern blot analyses for (B) *ΔcchA*, (C) *ΔmidA*, (D) *ΔyvcA*. (E) Schematic representation of the complementation assembly (F) The complementation was confirmed by PCR amplifying of a 2 kb fragment within each ORF.
(PPTX)

Figure S3 **Biofilm formation is decreased in the** ***ΔmidA*** **and** ***ΔyvcA*** **mutant strains.** (A) Biofilm formation evaluated by crystal violet absorbance at 570 nm. Data were expressed as the percentage of adhesion (expressed as 100% for the wild-type). Biomass formation as biofilm (B) or planktonic (C) growth. Statistical analysis was performed by using *t*-test (*, *p*<0.01).
(PPTX)

Table S1 **Accession numbers for all proteins used in the phylogenetic analyses.**
(XLS)

Table S2 **Primers used in this work.**
(DOCX)

File S1 **ARRIVE Checklist.**
(PDF)

## References

[1] Berridge MJ, Bootman MD, Llewelyn Roderick H (2003) Calcium Signalling: Dynamics, Homeostasis And Remodelling. Nature Reviews Molecular Cell Biology 4: 517–529. 12838335 10.1038/nrm1155

[2] Plattner H, Verkhratsky A (2013) Ca^2+^ signalling early in evolution–all but primitive. J Cell Sci 126: 2141–2150. 23729741 10.1242/jcs.127449

[3] Muller EM, Locke EG, Cunningham KW (2001) Differential Regulation of Two Ca^2+^ Influx Systems by Pheromone Signaling in *Saccharomyces cerevisiae* . Genetics 159: 1527–1538. 11779794 10.1093/genetics/159.4.1527 PMC1461924

[4] Yoshimoto H, Saltsman K, Gasch AP, Li HX, Ogawa N, et al (2002) Genome-wide analysis of gene expression regulated by the calcineurin/Crz1p signaling pathway in *Saccharomyces cerevisiae* . J Biol Chem 277: 31079–31088. 12058033 10.1074/jbc.M202718200

[5] Kraus PR, Heitman J (2003) Coping with stress: calmodulin and calcineurin in model and pathogenic fungi. Biochem Biophys Res Commun 311: 1151–1157. 14623301 10.1016/s0006-291x(03)01528-6

[6] da Silva Ferreira ME, Heinekamp T, Hartl A, Brakhage AA, Semighini CP, et al (2007) Functional characterization of the *Aspergillus fumigatus* calcineurin. Fungal Genet Biol 44: 219–230. 16990036 10.1016/j.fgb.2006.08.004

[7] Cavinder B, Trail F (2012) Role of Fig1, a Component of the Low-Affinity Calcium Uptake System, in Growth and Sexual Development of Filamentous Fungi. Eukaryotic Cell 11: 978–988. 22635922 10.1128/EC.00007-12 PMC3416067

[8] Wang S, Cao J, Liu X, Hu H, Shi J, et al (2012) Putative calcium channels CchA and MidA play the important roles in conidiation, hyphal polarity and cell wall components in *Aspergillus nidulans* . PLoS One 7: e46564. 23071589 10.1371/journal.pone.0046564 PMC3470553

[9] Fox DS, Heitman J (2002) Good fungi gone bad: the corruption of calcineurin. Bioessays 24: 894–903. 12325122 10.1002/bies.10157

[10] Cyert MS (2003) Calcineurin signaling in *Saccharomyces cerevisiae*: how yeast go crazy in response to stress. Biochem Biophys Res Commun 311: 1143–1150. 14623300 10.1016/s0006-291x(03)01552-3

[11] Stathopoulos-Gerontides A, Guo JJ, Cyert MS (1999) Yeast calcineurin regulates nuclear localization of the Crz1p transcription factor through dephosphorylation. Genes Dev 13: 798–803. 10197980 10.1101/gad.13.7.798 PMC316598

[12] Karababa M, Valentino E, Pardini G, Coste AT, Bille J, et al (2006) *CRZ1*, a target of the calcineurin pathway in *Candida albicans* . Mol Microbiol 59: 1429–1451. 16468987 10.1111/j.1365-2958.2005.05037.x

[13] Lew RR, Abbas Z, Anderca MI, Free SJ (2008) Phenotype of a mechanosensitive channel mutant, mid-1, in a filamentous fungus, *Neurospora crassa* . Eukaryot Cell 7: 647–655. 18296620 10.1128/EC.00411-07 PMC2292622

[14] Martin DC, Kim H, Mackin NA, Maldonado-Báez L, Evangelista CC Jr, et al (2011) New regulators of a high affinity Ca^2+^ influx system revealed through a genome-wide screen in yeast. J Biol Chem 286: 10744–10754. 21252230 10.1074/jbc.M110.177451 PMC3060525

[15] Groppi S, Belotti F, Brandão RL, Martegani E, Tisi R (2011) Glucose-induced calcium influx in budding yeast involves a novel calcium transport system and can activate calcineurin. Cell Calcium 49: 376–386. 21511333 10.1016/j.ceca.2011.03.006

[16] Harren K, Tudzynski B (2013) Cch1 and Mid1 are functionally required for vegetative growth under low-calcium conditions in the phytopathogenic ascomycete Botrytis cinerea. Eukaryot Cell 12: 712–724. 23475703 10.1128/EC.00338-12 PMC3647764

[17] Fischer M, Schnell N, Chattaway J, Davies P, Dixon G, et al (1997) The *Saccharomyces cerevisiae* CCH1 gene is involved in calcium influx and mating. FEBS Lett 419: 259–262. 9428646 10.1016/s0014-5793(97)01466-x

[18] Paidhungat M, Garrett S (1997) A homolog of mammalian, voltage-gated calcium channels mediates yeast pheromone-stimulated Ca^2+^ uptake and exacerbates the cdc1(Ts) growth defect. Mol Cell Biol 17: 6339–6347. 9343395 10.1128/mcb.17.11.6339 PMC232485

[19] Iida H, Nakamura H, Ono T, Okumura MS, Anraku Y (1994) MID1, a novel *Saccharomyces cerevisiae* gene encoding a plasma membrane protein, is required for Ca^2+^ influx and mating. Mol Cell Biol 14: 8259–8271. 7526155 10.1128/mcb.14.12.8259-8271.1994 PMC359365

[20] Ding X, Yu Q, Xu N, Wang Y, Cheng X, et al (2013) Ecm7, a regulator of HACS, functions in calcium homeostasis maintenance, oxidative stress response and hyphal development in *Candida albicans* . Fungal Genet Biol 57: 23–32. 23769872 10.1016/j.fgb.2013.05.010

[21] Muller EM, Mackin NA, Erdman SE, Cunningham KW (2003) Fig1p facilitates Ca^2+^ influx and cell fusion during mating of *Saccharomyces cerevisiae* . J Biol Chem 278: 38461–38469. 12878605 10.1074/jbc.M304089200

[22] Hong MP, Vu K, Bautos J, Gelli A (2010) Cch1 restores intracellular Ca^2+^ in fungal cells during ER stress. J Biol Chem 285: 10951–10958. 20123986 10.1074/jbc.M109.056218 PMC2856300

[23] Peiter E, Fischer M, Sidaway K, Roberts SK, Sanders D (2005) The *Saccharomyces cerevisiae* Ca^2+^ channel Cch1pMid1p is essential for tolerance to cold stress and iron toxicity. FEBS Lett 579: 5697–5703. 16223494 10.1016/j.febslet.2005.09.058

[24] Viladevall L, Serrano R, Ruiz A, Domenech G, Giraldo J, et al (2004) Characterization of the calcium-mediated response to alkaline stress in *Saccharomyces cerevisiae* . J Biol Chem 279: 43614–43624. 15299026 10.1074/jbc.M403606200

[25] Nguyen QB, Kadotani N, Kasahara S, Tosa Y, Mayama S, et al (2008) Systematic functional analysis of calcium-signalling proteins in the genome of the rice-blast fungus, *Magnaporthe oryzae*, using a highthroughput RNA-silencing system. Mol Microbiol 68: 1348–1365. 18433453 10.1111/j.1365-2958.2008.06242.x

[26] Cavinder B, Hamam A, Lew RR, Trail F (2011) Mid1, a mechanosensitive calcium ion channel, affects growth, development, and ascospore discharge in the filamentous fungus *Gibberella zeae* . Eukaryot Cell 10: 832–841. 21357477 10.1128/EC.00235-10 PMC3127676

[27] Hallen HE, Trail F (2008) The L-type calcium ion channel Cch1 affects ascospore discharge and mycelial growth in the filamentous fungus *Gibberella zeae* (anamorph *Fusarium graminearum*). Eukaryot Cell 7: 415–424. 18083828 10.1128/EC.00248-07 PMC2238158

[28] Bormann J, Tudzynski P (2009) Deletion of Mid1, a putative stretch activated calcium channel in *Claviceps purpurea*, affects vegetative growth, cell wall synthesis and virulence. Microbiology 155: 3922–3933. 19762439 10.1099/mic.0.030825-0

[29] Liu M, Du P, Heinrich G, Cox GM, Gelli A (2006) Cch1 mediates calcium entry in *Cryptococcus neoformans* and is essential in low-calcium environments. Eukaryotic Cell 5: 1788–1796. 16950930 10.1128/EC.00158-06 PMC1595334

[30] Teng JF, Goto R, Iida K, Kojima I, Iida H (2008) Ion-channel blocker sensitivity of voltage-gated calcium-channel homologue Cch1 in *Saccharomyces cerevisiae* . Microbiology 154: 3775–3781. 19047745 10.1099/mic.0.2008/021089-0

[31] Kmetzsch L, Staats CC, Rodrigues ML, Schrank A, Vainstein MH (2011) Calcium signaling components in the human pathogen: *Cryptococcus neoformans* . Commun Integr Biol 4: 186–187. 21655435 10.4161/cib.4.2.14271 PMC3104574

[32] Pittman JK (2011) Vacuolar Ca^(2+)^ uptake. Cell Calcium 50: 139–146. 21310481 10.1016/j.ceca.2011.01.004

[33] Pinchai N, Juvvadi PR, Fortwendel JR, Perfect BZ, Rogg LE, et al (2010) The *Aspergillus fumigatus* P-type Golgi apparatus Ca^2+^/Mn^2+^ ATPase PmrA is involved in cation homeostasis and cell wall integrity but is not essential for pathogenesis. Eukaryot Cell 9: 472–476. 20097742 10.1128/EC.00378-09 PMC2837981

[34] Palmer CP, Zhou XL, Lin J, Loukin SH, Kung C, et al (2001) A TRP homolog in *Saccharomyces cerevisiae* forms an intracellular Ca(2+)-permeable channel in the yeast vacuolar membrane. Proc Natl Acad Sci USA 98: 7801–7805. 11427713 10.1073/pnas.141036198 PMC35422

[35] Zhou XL, Batiza AF, Loukin SH, Palmer CP, Kung C, et al (2003) The transient receptor potential channel on the yeast vacuole is mechanosensitive. Proc Natl Acad Sci U S A 100: 7105–7110. 12771382 10.1073/pnas.1230540100 PMC165837

[36] Denis V, Cyert MS (2002) Internal Ca^(2+)^ release in yeast is triggered by hypertonic shock and mediated by a TRP channel. J Cell Biol 156: 29–34. 11781332 10.1083/jcb.200111004 PMC2173594

[37] Chang Y, Schlenstedt G, Flockerzi V, Beck A (2010) Properties of the intracellular transient receptor potential (TRP) channel in yeast, Yvc1. FEBS Lett 584: 2028–2032. 20035756 10.1016/j.febslet.2009.12.035

[38] Wang H, Liang Y, Zhang B, Zheng W, Xing L, et al (2011) Alkaline stress triggers an immediate calcium fluctuation in *Candida albicans* mediated by Rim101p and Crz1p transcription factors. FEMS Yeast Res 11: 430–439. 21457451 10.1111/j.1567-1364.2011.00730.x

[39] Yu Q, Wang F, Zhao Q, Chen J, Zhang B, et al (2013) A novel role of the vacuolar calcium channel Yvc1 in stress response, morphogenesis and pathogenicity of *Candida albicans* . Int J Med Microbiol S1438-4221(13)00195-1. 10.1016/j.ijmm.2013.11.022 24368068

[40] Greenberger PA (2002) Allergic bronchopulmonary aspergillosis. J Allergy Clin Immunol 110: 685–692. 12417875 10.1067/mai.2002.130179

[41] Dagenais TR, Keller NP (2009) Pathogenesis of *Aspergillus fumigatus* in Invasive Aspergillosis. Clin Microbiol Rev 22: 447–465. 19597008 10.1128/CMR.00055-08 PMC2708386

[42] Soriani FM, Malavazi I, da Silva Ferreira ME, Savoldi M, von Zeska Kress MR, et al (2008) Functional characterization of the *Aspergillus fumigatus* CRZ1 homologue, CrzA. Mol Microbiol 67: 1274–1291. 18298443 10.1111/j.1365-2958.2008.06122.x

[43] Mota Júnior AO, Malavazi I, Soriani FM, Heinekamp T, Jacobsen I, et al (2008) Molecular characterization of the *Aspergillus fumigatus* NCS-1 homologue, NcsA. Mol Genet Genomics 280: 483–495. 18830711 10.1007/s00438-008-0381-y

[44] Malavazi I, da Silva Ferreira ME, Soriani FM, Dinamarco TM, Savoldi M, et al (2009) Phenotypic analysis of genes whose mRNA accumulation is dependent on calcineurin in *Aspergillus fumigatus* . Fungal Genet Biol 46: 791–802. 19573616 10.1016/j.fgb.2009.06.009

[45] Dinamarco TM, Freitas FZ, Almeida RS, Brown NA, dos Reis TF, et al (2012) Functional characterization of an *Aspergillus fumigatus* calcium transporter (PmcA) that is essential for fungal infection. PLoS One 7: e37591. 22649543 10.1371/journal.pone.0037591 PMC3359301

[46] Cowen LE, Steinbach WJ (2008) Stress, drugs, and evolution: the role of cellular signaling in fungal drug resistance. Eukaryot Cell 7: 747–764. 18375617 10.1128/EC.00041-08 PMC2394973

[47] Yu Q, Ding X, Zhang B, Xu N, Jia C, et al (2014) Inhibitory effect of verapamil on *Candida albicans* hyphal development, adhesion and gastrointestinal colonization. FEMS Yeast Res (*in press*). 10.1111/1567-1364.12150 24650198

[48] Berridge MJ, Lipp P, Bootman MD (2000) The versatility and universality of calcium signaling. Nat Rev Mol Cell Biol 1: 11–21. 11413485 10.1038/35036035

[49] Steinbach WJ, Reedy JL, Cramer RA, Perfect JR Jr, Heitman J (2007) Harnessing calcineurin as a novel anti-infective agent against invasive fungal infections. Nat Rev Microbiol 5: 418–430. 17505522 10.1038/nrmicro1680

[50] Soriani FM, Malavazi I, Savoldi M, Espeso E, Dinamarco TM, et al (2010) Identification of possible targets of the *Aspergillus fumigatus* CRZ1 homologue, CrzA. BMC Microbiol 10: 12. 20078882 10.1186/1471-2180-10-12 PMC2818617

[51] Cramer RAJ, Perfect BZ, Pinchai N, Park S, Perlin DS, et al (2008) Calcineurin target *crzA* regulates conidial germination, hyphal growth, and pathogenesis of *Aspergillus fumigatus* . Eukaryot Cell 7: 1085–1097. 18456861 10.1128/EC.00086-08 PMC2446674

[52] Juvvadi PR, Fortwendel JR, Rogg LE, Burns KA, Randell SH, et al (2011) Localization and activity of the calcineurin catalytic and regulatory subunit complex at the septum is essential for hyphal elongation and proper septation in *Aspergillus fumigatus* . Mol Microbiol 82: 1235–1259. 22066998 10.1111/j.1365-2958.2011.07886.x PMC3225650

[53] Juvvadi PR, Gehrke C, Fortwendel JR, Lamoth F, Soderblom EJ, et al (2013) Phosphorylation of Calcineurin at a novel serine-proline rich region orchestrates hyphal growth and virulence in *Aspergillus fumigatus* . PLoS Pathog 9: e1003564. 23990785 10.1371/journal.ppat.1003564 PMC3749960

[54] Zelter A, Bencina M, Bowman BJ, Yarden O, Read ND (2004) A comparative genomic analysis of the calcium signaling machinery in *Neurospora crassa*, *Magnaporthe grisea*, and *Saccharomyces cerevisiae* . Fungal Genet Biol 41: 827–841. 15288019 10.1016/j.fgb.2004.05.001

[55] Rispail N, Soanes DM, Ant C, Czajkowski R, Grünler A, et al (2009) Comparative genomics of MAP kinase and calcium-calcineurin signalling components in plant and human pathogenic fungi. Fungal Genet Biol 46: 287–298. 19570501 10.1016/j.fgb.2009.01.002

[56] Ozeki-Miyawaki C, Moriya Y, Tatsumi H, Iida H, Sokabe M (2005) Identification of functional domains of Mid1, a stretch-activated channel component, necessary for localization to the plasma membrane and Ca^2+^ permeation. Exp Cell Res 311: 84–95. 16202999 10.1016/j.yexcr.2005.08.014

[57] Locke EG, Bonilla M, Liang L, Takita Y, Cunningham KW (2000) A homolog of voltage-gated Ca^2+^ channels stimulated by depletion of secretory Ca^2+^ in yeast. Mol Cell Biol 20: 6686–6694. 10958666 10.1128/mcb.20.18.6686-6694.2000 PMC86178

[58] Maruyama J, Kitamoto K (2007) Differential distribution of the endoplasmic reticulum network in filamentous fungi. FEMS Microbiol Lett 272: 1–7. 17517068 10.1111/j.1574-6968.2007.00758.x

[59] Iida K, Tada T, Iida H (2004) Molecular cloning in yeast by in vivo homologous recombination of the yeast putative α1 subunit of the voltage-gated calcium channel. FEBS Lett 576: 291–296. 15498550 10.1016/j.febslet.2004.09.021

[60] Yu Q, Wang H, Cheng X, Xu N, Ding X, et al (2012) Roles of Cch1 and Mid1 in morphogenesis, oxidative stress response and virulence in *Candida albicans* . Mycopathologia 174: 359–369. 22886468 10.1007/s11046-012-9569-0

[61] Ureshino RP, Rocha KK, Lopes GS, Trindade CB, Smaili SS (2014) Calcium signaling alterations, oxidative stress and autophagy in aging. Antioxid Redox Signal (*in press*). 10.1089/ars.2013.5777 24512092

[62] Yazgan O, Krebs JE (2012) Mitochondrial and nuclear genomic integrity after oxidative damage in *Saccharomyces cerevisiae* . Front Biosci 17: 1079–1093. 10.2741/3974 22201791

[63] Jiang H, Shen Y, Liu W, Lu L (2014) Deletion of the putative stretch-activated ion channel Mid1 is hypervirulent in *Aspergillus fumigatus* . Fungal Genet Biol 62: 62–70. 24239700 10.1016/j.fgb.2013.11.003

[64] Krishnan K, Ren Z, Losada L, Nierman WC, Lu LJ, et al (2014) Polysome profiling reveals broad translatome remodeling during endoplasmic reticulum (ER) stress in the pathogenic fungus *Aspergillus fumigatus* . BMC Genomics 15: 159. 24568630 10.1186/1471-2164-15-159 PMC3943501

[65] Kafer E (1977) Meiotic and mitotic recombination in *Aspergilllus* and its chromosomal aberrations. Adv Genet 19: 33–131. 327767 10.1016/s0065-2660(08)60245-x

[66] Colot HH, Park G, Turner GE, Ringelberg C, Crew CM, et al (2006) A highthroughput gene knockout procedure for *Neurospora* reveals functions for multiple transcription factors,. Proc Nat Acad Sci USA 103: 10352–10357. 16801547 10.1073/pnas.0601456103 PMC1482798

[67] Schiestl RH, Gietz RD (1989) High efficiency transformation of intact yeast cells using single stranded nucleic acids as a carrier. Curr Genet 16: 339–346. 2692852 10.1007/BF00340712

[68] Goldman GH, dos Reis Marques E, Duarte Ribeiro DC, de Souza Bernardes LA, Quiapin AC, et al (2003) Expressed sequence tag analysis of the human pathogen *Paracoccidioides brasiliensis* yeast phase: identification of putative homologues of *Candida albicans* virulence and pathogenicity genes. Eukaryot Cell 2: 34–48. 12582121 10.1128/EC.2.1.34-48.2003 PMC141168

[69] Herrera-Estrella A, Goldman GH, Van Montagu M (1990) High-efficiency transformation system for the biocontrol agents, *Trichoderma* spp. Mol Microbiol 4: 839–43. 2388561 10.1111/j.1365-2958.1990.tb00654.x

[70] Teepe AG, Loprete DM, He Z, Hoggard TA, Hill TW (2007) The protein kinase C orthologue PkcA plays a role in cell wall integrity and polarized growth in *Aspergillus nidulans* . Fungal Genet Biol 44: 554–562. 17118679 10.1016/j.fgb.2006.10.001

[71] Shoji J-Y, Arioka M, Kitamoto K (2006) Vacuolar membrane dynamics in the filamentous fungus *Aspergillus oryzae* . Eukaryot Cell 5: 411–421. 16467481 10.1128/EC.5.2.411-421.2006 PMC1405889

[72] Mowat E, Butcher J, Lang S, Williams C, Ramage G (2007) Development of a simple model for studying the effects of antifungal agents on multicellular communities of *Aspergillus fumigatus* . J Med Microbiol 56: 1205–1212. 17761484 10.1099/jmm.0.47247-0

[73] Shopova I, Bruns S, Thywissen A, Kniemeyer O, Brakhage AA, et al (2013) Extrinsic extracellular DNA leads to biofilm formation and colocalizes with matrix polysaccharides in the human pathogenic fungus *Aspergillus fumigatus* . Front Microbiol 4: 141. 23760756 10.3389/fmicb.2013.00141 PMC3674311

[74] Semighini CP, Marins M, Goldman MHS, Goldman GH (2002) Quantitative analysis of the relative transcript levels of ABC transporter *Atr* genes in *Aspergillus nidulans* by real-time reverse transcription-PCR assay. Appl Environ Microbiol 68: 1351–1357. 11872487 10.1128/AEM.68.3.1351-1357.2002 PMC123782

