# Supplementary material for: The Involvement of the Mid1/Cch1/Yvc1 Calcium Channels in Aspergillus fumigatus Virulence
Source: PLoS One. 2014 Aug 1;9(8):e103957. doi: 10.1371/journal.pone.0103957 (PMC4118995; doi:10.1371/journal.pone.0103957)
Supplement: Table S2 — Primers used in this work. (DOCX) [file pone.0103957.s005.docx]

Table S2: Primers used in this work

| **Name** | **5’-3’ Sequence** |
| --- | --- |
| pRS426_5UTR yvc_F | GTAACGCCAGGGTTTTCCCAGTCACGACGGATTGATTGTCGAAGAACTCGCCTG |
| 5UTR yvc_pyrG_R | CGCATCAGTGCCTCCTCTCAGACAGAATCGAGCAAGATTAAGTTCCCACACTAA |
| pyrG_3UTR yvc_F | GGTGAAGAGCATTGTTTGAGGCGAATTCGCGGAGTAGGATTTATGTATGGGACAG |
| 3UTR yvc_ pRS426_R | GCGGTTAACAATTTCTCTCTGGAAACAGCCAGCAGGGCACGAGATCAGTTA |
| pRS426_5UTR mid_F | GTAACGCCAGGGTTTTCCCAGTCACGACGAGTTTTGGGCCTGTCATCCGAGA |
| 5UTR mid_pyrG_R | GTGCCTCCTCTCAGACAGAATCAACCAACTGGGCTCCGAAGGACG |
| pyrG_3UTR mid_F | GAGCATTGTTTGAGGCGAATTCATGCATTCTCTTTTTCAGGATTC |
| 3UTR mid_ pRS426_R | GCGGTTAACAATTTCTCTCTGGAAACAGCTCCGCCATTAAAGATGCCATTGC |
| pRS426_5UTR cch_F | GTAACGCCAGGGTTTTCCCAGTCACGACGCTACCTCGTGTCTTGATCTATCCG |
| 5UTR cch_pyrG_R | GTGCCTCCTCTCAGACAGAATCGTAGGCAGGTATAATGCGCTAGCC |
| pyrG_3UTR cch_F | GAGCATTGTTTGAGGCGAATTCGCAATAGAAACGCAAGCAATGTTG |
| 3UTR cch_ pRS426_R | GCGGTTAACAATTTCTCTCTGGAAACAGCCTCTGAAACCGTCTCACCCAA |
| yvc pRS426 5Fw | GTAACGCCAGGGTTTTCCCAGTCACGACGGGGCTTCGGTTATGACACGAA |
| yvc SPACER GFP Rv | AGTTCTTCTCCTTTACTCATTCCCCGTGTTCCCTCCGCGTCACCCTGATT |
| Spacer GFP Fw | GGAACACGGGGAATGAGTAAAGGAGAAGAACT |
| GFP VE3’ AF | CTCAGACAGAATACGCCAAGCTTGCATGC |
| GFP pyrG Fw | GCATGCAAGCTTGGCGTATTCTGTCTGAGAGGAGGC |
| pyrG Rv | GAATTCGCCTCAAACAATGCTCTTCACC |
| Afu yvc 3Fw | GCATTGTTTGAGGCGAATTCTGTATGGGACAGTTGTCTTGG |
| Afu yvc 3Rv | GCGGATAACAATTTCACACAGGAAACAGTGAGATGCGGGCATTATGACC |
| AscI mid Fw | GGCGCGCC ATGCAACTATCCATGCGGACT |
| PacI mid Rv | CCTTAATTAAGGCTAGGCGTCCACCCTGAGAGC |
| AscI cch Fw | GGCGCGCCATGGCTTCGAATAGCCACAAC |
| PacI cch Rv | CCTTAATTAAGGTTACGGTTGACTACCGTTCGA |
| Afu B tubulin Fw Sybr | ATATGTTCCTCGTGCCGTTC |
| Afu B tubulin Rv Sybr | GAGAGAGTGGGTGACCTGGA |
| Afu cch Fw Sybr | CGTTCCTTGTCTCCTTCAGC |
| Afu cch Rv Sybr | ATTCTTGTGCTGTTCCTCCC |
| Afu mid Fw Sybr | AAGCCAGCAAACTGTCAGGT |
| Afu mid Rv Sybr | GAGTGACTCCGAGGTAGACA |
